# Characteristics Associated with Ventricular Tachyarrhythmias and Their Prognostic Impact in Heart Failure with Mildly Reduced Ejection Fraction

**DOI:** 10.3390/jcm13092665

**Published:** 2024-05-01

**Authors:** Alexander Schmitt, Michael Behnes, Jonas Rusnak, Muharrem Akin, Marielen Reinhardt, Noah Abel, Jan Forner, Julian Müller, Kathrin Weidner, Mohammad Abumayyaleh, Ibrahim Akin, Tobias Schupp

**Affiliations:** 1 First Department of Medicine, Section for Invasive Cardiology, University Medical Centre Mannheim, Medical Faculty Mannheim, Heidelberg University, 68167 Mannheim, Germany; cw224@stud.uni-heidelberg.de (A.S.); michael.behnes@umm.de (M.B.);; 2 Department of Cardiology, Angiology and Pneumology, University Hospital Heidelberg, 69047 Heidelberg, Germany; 3 Department of Cardiology, St. Josef-Hospital, Ruhr-Universität Bochum, 44791 Bochum, Germany; 4 Department of Cardiology, Faculty of Medicine, University Heart Center Freiburg-Bad Krozingen, University of Freiburg, 79106 Freiburg im Breisgau, Germany

**Keywords:** heart failure with mildly reduced ejection fraction, HFmrEF, ventricular tachyarrhythmias, ventricular tachycardia, sudden cardiac death

## Abstract

**Background**: The occurrence of ventricular tachyarrhythmias represents an established risk factor of mortality in heart failure (HF). However, data concerning their prognostic impact in heart failure with mildly reduced ejection fraction (HFmrEF) is limited. Therefore, the present study aims to investigate patient characteristics associated with ventricular tachyarrhythmias and their prognostic impact in patients with HFmrEF. **Methods**: Consecutive patients hospitalized with HFmrEF (i.e., left ventricular ejection fraction 41–49% and signs and/or symptoms of HF) were retrospectively included at one institution from 2016 to 2022. The prognosis of patients with HFmrEF and different types of ventricular tachyarrhythmias (i.e., non-sustained ventricular tachycardia (nsVT), sustained VT (sVT), and ventricular fibrillation (VF) was investigated for the primary endpoint of long-term all-cause mortality at 30 months. Secondary endpoints included in-hospital all-cause mortality and long-term HF-related rehospitalization at 30 months. **Results**: From a total of 2184 patients with HFmrEF, 4.4% experienced ventricular tachyarrhythmias (i.e., 2.0% nsVT, 0.7% sVT, and 1.6% VF). The occurrence of nsVT was associated with higher New York Heart Association (NYHA) functional class, whereas the incidence of sVT/VF was associated with acute myocardial infarction and ischemic heart disease. However, nsVT (25.0%; HR = 0.760; 95% CI 0.419–1.380; *p* = 0.367) and sVT/VF (28.8%; HR = 0.928; 95% CI 0.556–1.549; *p* = 0.776) were not associated with a higher risk of long-term all-cause mortality compared to patients with HFmrEF without ventricular tachyarrhythmias (31.5%). In-hospital cardiovascular mortality was more frequently observed in patients with HFmrEF and sVT/VF compared to those with HFmrEF but without sustained ventricular tachyarrhythmias (7.7% vs. 1.5%; *p* = 0.004). Finally, the risk of rehospitalization for worsening HF was not affected by the presence of ventricular tachyarrhythmias. **Conclusions**: The occurrence of ventricular tachyarrhythmias in patients hospitalized with HFmrEF was low and not associated with long-term prognosis.

## 1. Introduction

Sudden cardiac death (SCD) remains a challenging manifestation of cardiovascular disease and accounts for up to 50% of all cardiovascular deaths. Up to half of these deaths occur as the first event of cardiac disease [1,2]. Notably, SCD is related to coronary artery disease (CAD) in almost 80% of cases. As CAD represents one of the most common risk factors for the occurrence of ventricular tachyarrhythmias, a large proportion of SCD can be regarded as sudden arrhythmic death (SAD) [3,4,5,6,7]. Despite the establishment of many preventative approaches to reduce SCD through implantable cardioverter defibrillators (ICD) or community-based cardiopulmonary resuscitation (CPR) programs, SCD remains responsible for 15–20% of all deaths in Western countries [5,8]. Due to the lack of evidence regarding ventricular tachyarrhythmias in specific patient cohorts, reliable identification of patients at risk remains complex [9,10].

Several studies have suggested that heart failure (HF) may represent a major risk factor for ventricular tachycardia (VT), ventricular fibrillation (VF), and therefore SAD through complex cardiac remodeling with changes in the electrical function of the heart (e.g., alteration in Ca^2+^-homeostasis, action potential prolongation, and functional downregulation of K^+^-currents) [11,12,13]. Although a relevant part of SAD occurs in presumably healthy individuals, left ventricular ejection fraction (LVEF) remains one of the most important predictors of SAD and guides potentially life-saving supply with ICD in patients with HF [14,15,16]. However, studies investigating HF and ventricular tachyarrhythmias have primarily focused on patients with reduced ejection fraction (HFrEF), but only rarely on mildly reduced ejection fraction (HFmrEF; LVEF 41–49%) or preserved ejection fraction (HFpEF), which—by now—are underrepresented in clinical studies [17,18,19,20]. Due to the scarcity of data on ventricular tachyarrhythmias in these cohorts, their true incidence and mortality burden in patients with HFmrEF and HFpEF remain unknown. As a result, there is no evidence about the value of ICD implantation in patients with mildly reduced and preserved LVEF to prevent SAD, since current guideline recommendations are based on randomized controlled trials (RCT) that were restricted to patients with LVEF <40% [21,22,23,24,25]. In the prospective, multicenter VIP-HF study [26], a small combined HFmrEF/HFpEF cohort of 113 patients was investigated with implantable loop recorders to detect incident arrhythmias. In accordance with another contemporary investigation [27], relatively low rates of ventricular tachyarrhythmias were observed in patients with HFmrEF/HFpEF, as only one patient suffered from sVT (0.6/100 person-years), whereas 16 patients experienced nsVT (11.5/100 person-years) during a median follow-up of 657 days [26]. However, it must be considered that the study only recruited less than half of the intended sample size (n = 250) due to slow inclusion rates. The problem of relatively low event rates regarding ventricular tachyarrhythmias in patients with HFmrEF/HFpEF also poses an important difficulty that has resulted in the withdrawal of trials in these patient cohorts, such as the PROFID-Preserved trial [28]. Moreover, accurately documenting ventricular tachyarrhythmias in HFmrEF patients may pose a greater challenge compared to HFrEF, primarily because a lower percentage of HFmrEF patients have continuous rhythm monitoring through devices such as an ICD. This further underscores the value of data on ventricular tachyarrhythmia in patients with LVEF >40% and should emphasize the persistent necessity for future studies and the importance of the present study.

In addition, data concerning the implications of non-sustained VT (nsVT) in patients with HF remain heterogeneous. While some studies suggested that nsVT may be associated with worse clinical outcomes (e.g., all-cause mortality or ICD shocks) in patients with HF [17,29,30,31,32], others suggested the occurrence of nsVT was not associated with patient outcomes [33,34]. 

In general, data concerning predictors and the prognostic impact of nsVT as well as VT and VF in the patient cohort of HFmrEF remains limited [9,10]. Therefore, the present study aims to identify (1) patients’ characteristics associated with the occurrence of nsVT, sVT, and VF and (2) the prognostic role of ventricular tachyarrhythmias within a large-scaled retrospective registry of patients hospitalized with HFmrEF.

## 2. Materials and Methods

### 2.1. Study Patients, Design, and Data Collection

For the present study, all consecutive patients hospitalized with HFmrEF at one institution were included from January 2016 to December 2022. Using the electronic hospital information system, all relevant clinical data related to the index event were documented, such as baseline characteristics, vital signs on admission, prior medical history, prior medical treatment, length of index hospital and intensive care unit (ICU) stay, laboratory values, data derived from all non-invasive or invasive cardiac diagnostics and device therapies, such as echocardiographic data, coronary angiography, and data being derived from prior or newly implanted cardiac devices. Every re-visit to the outpatient clinic and rehospitalizations related to HF and adverse cardiac events were documented until the end of the year 2022. The university medical center covers a general emergency department for emergency admission of traumatic, surgical, neurological, and cardiovascular conditions. Interdisciplinary consultation is an inbuilt feature of this 24/7 service and connects to a stroke unit, four ICUs with extracorporeal life support, and a chest pain unit to alleviate rapid triage of patients. The cardiologic department itself includes a 24 h catheterization laboratory, an electrophysiologic laboratory, a hybrid operating room, and telemetry units. Furthermore, the medical center is a certified HF unit. 

The present study derived from the “Heart Failure With Mildly Reduced Ejection Fraction Registry” (HARMER), representing a retrospective single-center registry including consecutive patients with HFmrEF hospitalized at the University Medical Center Mannheim (UMM), Germany (clinicaltrials.gov identifier: NCT05603390). The registry was carried out according to the principles of the Declaration of Helsinki and was approved by the Medical Ethics Committee II of the Medical Faculty Mannheim, University of Heidelberg, Germany (ethical approval code: 2022-818).

### 2.2. Inclusion and Exclusion Criteria

All consecutive patients ≥ 18 years of age hospitalized with HFmrEF at one institution were included. All included patients underwent at least one standardized transthoracic echocardiography at the cardiologic department at index hospitalization, where the diagnosis of HFmrEF was assessed. The diagnosis of HFmrEF was determined retrospectively according to the “2021 European Society of Cardiology (ESC) guidelines for the diagnosis and treatment of acute and chronic HF” [14]. Accordingly, all patients with LVEF 41–49% and symptoms and/or signs of HF were included. The presence of elevated amino-terminal prohormone of brain natriuretic peptide (NT-proBNP) levels and other evidence of structural heart disease were considered to make the diagnosis more likely but were not mandatory for the diagnosis of HFmrEF. Standardized transthoracic echocardiography was performed by cardiologists during routine clinical care in accordance with current European guidelines [35]. Accordingly, only patients with sufficient hemodynamic stability to undergo standard echocardiographic assessment of LVEF were included. Diastolic dysfunction was determined through echocardiographic evaluation by measurement of transmitral flow parameters including the early (E) and late (A) diastolic filling velocities, the E/A ratio, and the E deceleration time from an apical four-chamber view with conventional pulsed wave Doppler [36,37]. Finally, all echocardiographic examinations and reports were re-assessed post hoc by two independent cardiologists blinded to the final data analysis. In cases of ambiguous findings or documentation, echocardiographic source data was re-assessed in individual cases based on the available Digital Imaging and Communications in Medicine (DICOM) files. All patients <18 years of age were excluded. No other exclusion criteria were applied.

### 2.3. Risk Stratification

For the present study, patients with at least one episode of nsVT or sVT/VF during the index admission were compared to patients without ventricular tachyarrhythmias. NsVT, sVT, and VF were defined according to current European guidelines [9]. Accordingly, sVT was defined by a duration of ≥30 s or causing hemodynamic collapse within 30 s, while nsVT was defined by a duration of <30 s accompanied by a wide QRS complex (≥120 ms) at a heart rate of >100 beats per minute (bpm) [9]. Furthermore, VF was defined as a chaotic rhythm with undulations that were irregular in timing and morphology, without discrete QRS complexes on the surface electrocardiogram (ECG) and a ventricular rate of >300 bpm [9]. Ventricular tachyarrhythmias were documented by standard 12-lead ECG, ECG telemetry, ICD, or, in case of an unstable course or during resuscitation, by external defibrillator monitoring. In cases of prolonged instability, additional intravenous anti-arrhythmic drugs were administered during CPR. Patients with episodes of nsVT and sVT/VF were included in the sVT/VF group. The prognostic impact of ventricular tachyarrhythmias was also investigated in patient subgroups stratified by the incidence of acute myocardial infarction (AMI) during the index admission (i.e., patients with or without AMI).

### 2.4. Study Endpoints

The primary endpoint was long-term all-cause mortality. Long-term was defined as the median time of clinical follow-up in months. Secondary endpoints comprised in-hospital all-cause mortality, which was further stratified as cardiovascular and non-cardiovascular as well as all-cause mortality at 12 months of follow-up. Further secondary endpoints included rehospitalization for worsening HF, cardiac rehospitalization, AMI, stroke, coronary revascularization, and major adverse cardiac and cerebrovascular events (MACCEs) at long-term follow-up. All-cause mortality was documented using the electronic hospital information system and by directly contacting state resident registration offices (“Bureau of Mortality Statistics”). Identification of patients was verified by place of name, surname, day of birth, and registered living address. HF-related hospitalization was defined as a rehospitalization due to worsening HF requiring intravenous diuretic therapy. HF-related rehospitalization comprises patients with hospitalization due to worsening HF as the primary cause or as a result of another cause but associated with worsening HF at the time of admission, or as a result of another cause but complicated by worsening HF during its cause. Cardiac rehospitalization was defined as rehospitalization due to a primary cardiac condition, including worsening HF, AMI, coronary revascularization, and symptomatic atrial or ventricular arrhythmias. MACCE was described as a composite of all-cause mortality, coronary revascularization, non-fatal AMI, and non-fatal stroke.

### 2.5. Statistical Methods

Quantitative data is presented as median and interquartile range (IQR). Deviations from a Gaussian distribution were tested by the Kolmogorov–Smirnov test. The three groups (i.e., none, nsVT, and sVT/VF) were compared using the Kruskal–Wallis test. In the case of a statistically significant result (*p* ≤ 0.05), Dunn’s tests have been performed for pairwise comparisons (i.e., Mann–Whitney U-tests with Bonferroni correction in order to control the type I error). Qualitative data is presented as absolute and relative frequencies and were compared using the Chi-square test or the Fisher’s exact test, as appropriate. Pairwise comparisons for these factors have also been performed using the Bonferroni correction. Accordingly, the α-significance level for the three pairwise comparisons was adjusted to *p* ≤ 0.05/3. Logistic regression models were used to identify patient characteristics associated with the occurrence of nsVT and sVT/VF. Kaplan–Meier analyses were performed comparing patients with and without ventricular arrhythmias (i.e., nsVT and sVT/VF), and univariable hazard ratios (HRs) were given together with 95% confidence intervals (CIs). The prognostic impact of nsVT and sVT/VF was thereafter investigated within multivariable Cox regression models. Only variables with *p* ≤ 0.1 in univariable analyses were incorporated in the multivariable Cox regression model. Spearman’s rank correlation coefficients and variance-inflation factors were calculated to test for collinearity among the variables used for multivariable analyses. Results of all other statistical tests were considered significant for *p* ≤ 0.05. SPSS (Version 28, IBM, Armonk, NY, USA) was used for all statistical analyses.

## 3. Results

### 3.1. Study Population

From 2016 to 2022, 2228 consecutive patients with HFmrEF were included in the HARMER registry. The lost to follow-up rate with regard to all-cause mortality was 1.97% resulting in a final study cohort of 2184 patients with HFmrEF. Median follow-up time was 30 months (i.e., long-term follow-up). The overall incidence of nsVT was 2.0% (n = 44) and sVT/VF 2.4% (n = 16 with sVT, n = 27 with VF, and n = 9 with both sVT + VF). Patients with sVT/VF were younger than those without ventricular tachyarrhythmias or nsVT (68 years vs. 76 and 74 years, respectively; *p* = 0.003) (Table 1). Patients experiencing nsVT and sVT/VF during the index admission had higher rates of prior sVT/VF (11.4% and 15.4% vs. 2.5%; *p* = 0.001 for both comparisons). An ICD implantation prior to the index admission was significantly more frequent in patients with sVT/VF compared to the non-arrhythmic group (13.5% vs. 1.6%; *p* = 0.001). No relevant differences in cardiovascular risk factors were observed between all three groups, except for a higher prevalence of hyperlipidemia in the nsVT and sVT/VF groups (45.5% and 40.4% vs. 29.7%; *p* = 0.022). In comparison to the cohort without ventricular tachyarrhythmias, a significantly higher proportion of patients was admitted with ST-segment elevation myocardial infarction (STEMI) in the nsVT and sVT groups (7.2% vs. 25.0% and 28.8%; *p* ≤ 0.001). Non-STEMI (NSTEMI) was also more common in the sVT group than in patients without ventricular tachyarrhythmias (23.1% vs. 12.3%). Implantation of cardiac defibrillators during the index admission was rare in the entire study cohort but more frequent in patients with nsVT and sVT/VF (*p* ≤ 0.001 for all comparisons with the non-arrhythmic group).

Additional HF-related and procedural data is presented in Table 2. There was a high prevalence of ischemic heart disease (IHD) in nsVT (70.5%) and sVT/VF (88.5%) compared to the non-arrhythmic group (56.6%). Accordingly, coronary angiography was performed significantly more often in the nsVT and sVT/VF groups compared to patients without ventricular tachyarrhythmias (63.6% and 88.5% vs. 39.6%; *p* ≤ 0.004) with the highest rate of PCI in the sVT/VF group (71.7%). In addition, coronary chronic total occlusions (CTOs) were highly prevalent in the sVT/VF group compared to the nsVT and non-arrhythmic groups (34.8% vs. 7.1% and 11.5%; *p* ≤ 0.007). A statistical trend towards higher estimated glomerular filtration rates was observed in the arrhythmia groups (79 mL/min in both groups vs. 65 mL/min). Additional differences in baseline laboratory values include a higher platelet count (269 × 10^9^/L vs. 217 and 225 × 10^9^/L; *p* ≤ 0.007) and lower high-density lipoprotein (HDL) cholesterol levels (37 mg/dL vs. 44 and 42 mg/dL) in the sVT/VF group in comparison to patients with nsVT or without ventricular tachyarrhythmias. Within the sVT/VF group, angiotensin-converting enzyme (ACE) inhibitor (68.6% vs. 43.2% and 49.9%; *p* ≤ 0.023) and beta-blocker (100% vs. 88.6% and 76.7%; *p* ≤ 0.016) therapy at discharge were also significantly more frequent. Finally, statins were prescribed more often in the groups of nsVT and sVT/VF compared to the non-arrhythmic group (81.8% and 81.3% vs. 67.8%), although not reaching statistical significance in the pairwise comparisons. Supplementary data regarding ventricular arrhythmias in the study cohort are presented in Table 3.

### 3.2. Patient Characteristics Associated with nsVT and sVT/VF in Patients with HFmrEF

**Figure 1:**
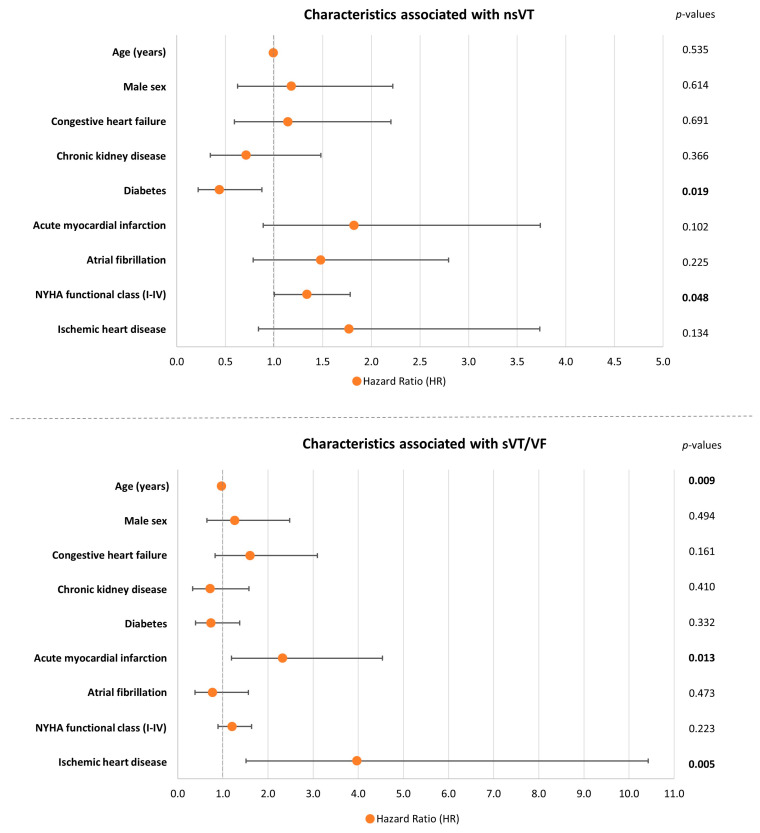
Forest plots demonstrating logistic regression analyses to identify patient characteristics associated with nsVT (**upper panel**) and sVT/VF (**lower panel**) with corresponding hazard ratios (scatter points) and 95% confidence intervals (error bars). Level of significance *p* ≤ 0.05. Bold type indicates statistical significance.

Figure 1 displays logistic regression analyses to identify patient characteristics associated with the occurrence of nsVT and sVT/VF. The incidence of nsVT was associated with higher NYHA functional class (HR = 1.338; 95% CI 1.003–1.784; *p* = 0.048), whereas patients with comorbid diabetes had a lower risk of nsVT (HR = 0.438; 95% CI 0.219–0.875; *p* = 0.019) (Figure 1, upper panel). Furthermore, the occurrence of sVT/VF was associated with AMI (HR = 2.326; 95% CI 1.192–4.539; *p* = 0.013) and IHD (HR = 3.973; 95% CI 1.514–10.426; *p* = 0.005), while advancing age was associated with a lower risk of sVT/VF (HR = 0.971; 95% CI 0.950–0.993; *p* = 0.009) (Figure 1, lower panel).

### 3.3. Prognostic Impact of nsVT and sVT/VF

**Figure 2:**
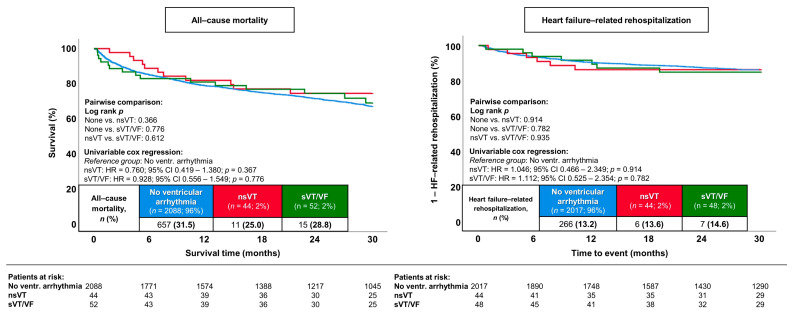
Prognostic impact of nsVT and sVT/VF on the risk of long-term all-cause mortality (**left panel**) and HF-related rehospitalization (**right panel**) within the entire study cohort.

The occurrence of nsVT (HR = 0.760; 95% CI 0.419–1.380; *p* = 0.367) and sVT/VF (HR = 0.928; 95% CI 0.556–1.549; *p* = 0.776) did not impact patients’ prognosis with regard to the primary endpoint of long-term all-cause mortality when compared to patients with HFmrEF who did not experience ventricular tachyarrhythmias (25.0% and 28.8% vs. 31.5%; log-rank *p* ≥ 0.366) (Figure 2, left panel). Similar results were observed for the secondary endpoint of long-term HF-related rehospitalization (log-rank *p* ≥ 0.782) (Figure 2, right panel). Regarding additional secondary endpoints and follow-up data, the duration of ICU stay was higher in the nsVT and sVT/VF groups (2 days and 1 day vs. 0 days; *p* ≤ 0.001 for all comparisons). However, no significant differences in the other secondary endpoints were observed, with the exception that in-hospital cardiovascular mortality was more frequently observed in patients with HFmrEF and sVT/VF compared to those with HFmrEF but without ventricular tachyarrhythmias (7.7% vs. 1.5%; HR = 4.587; 95% CI 1.618–13.003; *p* = 0.004) (Table 4).

### 3.4. Multivariable Cox Regression Analyses

**Figure 3:**
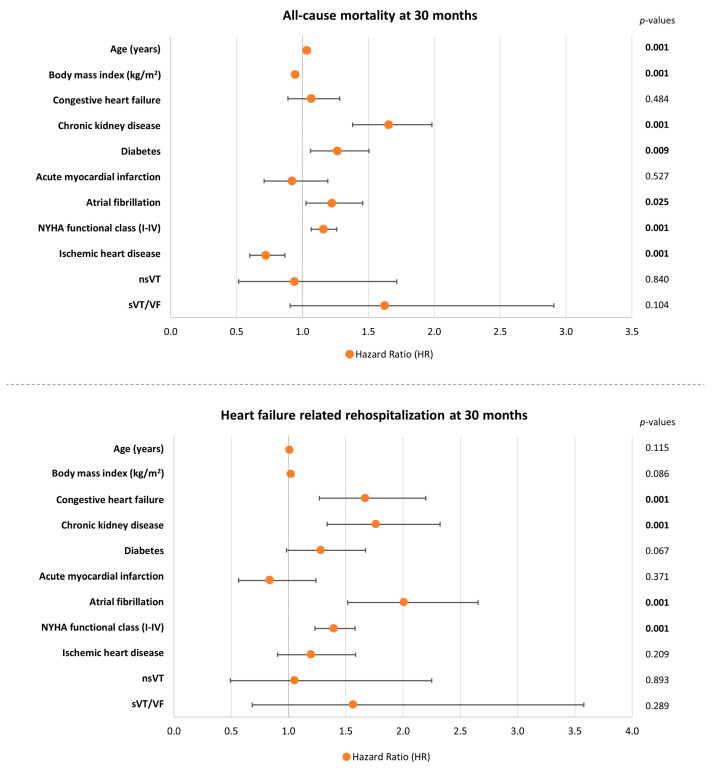
Forest plots demonstrating multivariable Cox regression analyses within the entire study cohort regarding the risk of long-term all-cause mortality (**upper panel**) and HF-related rehospitalization (**lower panel**). Level of significance *p* ≤ 0.05. Bold type indicates statistical significance.

After multivariable adjustment age (HR = 1.034; *p* = 0.001), chronic kidney disease (CKD; HR = 1.645; *p* = 0.001), diabetes mellitus (HR = 1.264; *p* = 0.009), atrial fibrillation (HR = 1.223; *p* = 0.025), and NYHA functional class (HR = 1.159; *p* = 0.001) were associated with a higher risk of long-term all-cause mortality in the entire study cohort of patients with HFmrEF. However, nsVT (HR = 0.940; 95% CI 0.515–1.715; *p* = 0.840) and sVT/VF (HR = 1.623; 95% CI 0.906–2.908; *p* = 0.104) were not associated with the risk of long-term all-cause mortality. In addition, body mass index (BMI, HR = 0.945; *p* = 0.001) and IHD (HR = 0.721; *p* = 0.001) were predictive of a lower risk of mortality (Figure 3, upper panel). In line with the primary endpoint, nsVT (HR = 1.053; 95% CI 0.493–2.250; *p* = 0.893) and sVT/VF (HR = 1.564; 95% CI 0.684–3.579; *p* = 0.289) were not associated with long-term HF-related rehospitalization. Nonetheless, prior congestive HF (HR = 1.669; *p* = 0.001), CKD (HR = 1.762; *p* = 0.001), atrial fibrillation (HR = 2.007; *p* = 0.001), and NYHA functional class (HR = 1.395; *p* = 0.001) were predictive of a higher risk of HF-related rehospitalizations at the long-term follow-up of 30 months (Figure 3, lower panel). To assess collinearity among the parameters included in the multivariable Cox regression model, Spearman’s rank correlation coefficients and variance inflation factors were calculated. The results are displayed in Supplementary Tables S1 and S2. Additionally, univariable analyses of the individual parameters incorporated in the multivariable Cox regression model are presented in Supplementary Table S3.

### 3.5. Prognostic Impact of nsVT and sVT/VF in Subgroups with or without AMI during the Index Admission

**Figure 4:**
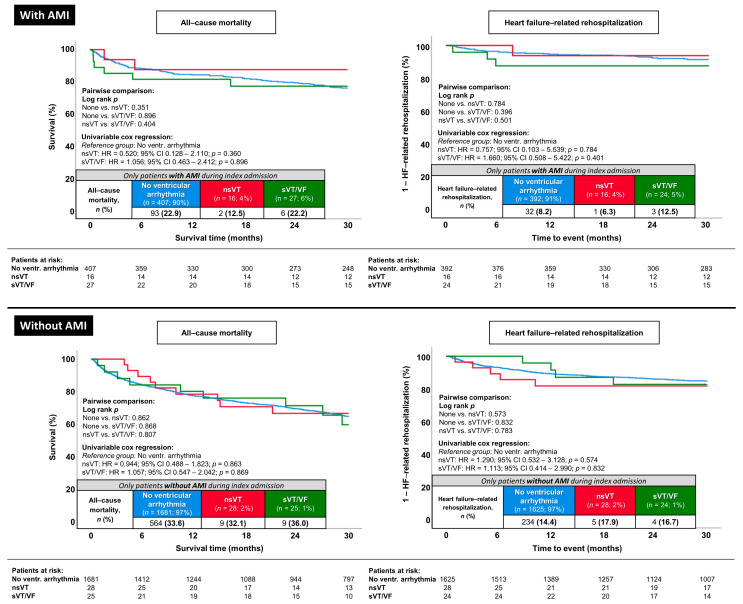
Prognostic impact of nsVT and sVT/VF on the risk of long-term all-cause mortality (**left column**) and HF-related rehospitalization (**right column**) stratified by the incidence of acute myocardial infarction (**upper panels**, AMI/**lower panels**, non-AMI) during the index hospitalization.

**Figure 5:**
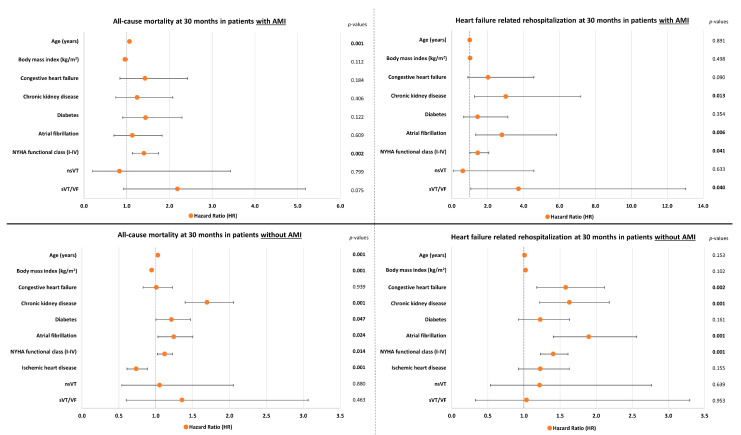
Forest plots demonstrating multivariable Cox regression analyses regarding the risk of long-term all-cause mortality (**left column**) and HF-related rehospitalization (**right column**) within subgroups stratified by the incidence of acute myocardial infarction (**upper panels**, AMI/**lower panels**, non-AMI) during the index admission. Level of significance *p* ≤ 0.05. Bold type indicates statistical significance.

As demonstrated in Figure 4, the occurrence of nsVT and sVT/VF also did not impact long-term all-cause mortality (log-rank *p* ≥ 0.351 for all comparisons) or HF-related rehospitalization (log-rank *p* ≥ 0.396 for all comparisons) in analyses selectively investigating subgroups stratified by the incidence of AMI during the index admission. Furthermore, multivariable Cox regression analyses were performed in these subgroups (i.e., AMI and non-AMI subgroups; Figure 5). Similar to the results observed in the entire study population (3.4), nsVT and sVT/VF were also not associated with the risk of long-term all-cause mortality when performing multivariable adjustment in subgroups exclusively composed of HFmrEF patients with (nsVT: *p* = 0.799 and sVT/VF: *p* = 0.075) or without AMI (nsVT: *p* = 0.880 and sVT/VF: *p* = 0.463) during the index admission (Figure 5, left column). In addition, nsVT and sVT/VF were not associated with long-term HF-related rehospitalization in the subgroup without AMI. However, analysis in the subgroup of patients with AMI during the index admission showed a higher risk of HF-related rehospitalization in patients experiencing sVT/VF (HR = 3.716; 95% CI 1.060–13.030; *p* = 0.040) (Figure 5, right column).

## 4. Discussion

The present study investigates patients’ characteristics associated with ventricular tachyarrhythmias (i.e., nsVT, VT, and VF) and their prognostic impact in patients with HFmrEF using a large retrospective cohort of 2184 patients hospitalized with HFmrEF. NsVT and sVT/VF within the index hospitalization occurred in 2.0% (n = 44) and 2.4% (n = 52) of patients, respectively. IHD was the leading etiology of HF within the entire cohort and was associated with the occurrence of sVT/VF, specifically in the setting of AMI. The incidence of nsVT was associated with higher NYHA functional class. In addition, diabetes and higher age were associated with a lower risk of nsVT and sVT/VF, respectively. The occurrence of both nsVT and sVT/VF was not associated with the primary endpoint of long-term all-cause mortality or most other secondary endpoints such as long-term HF-related rehospitalization or MACCE. However, the highest rate of in-hospital cardiovascular mortality was observed in the group with sVT/VF. Subgroup analyses of the prognostic impact of ventricular tachyarrhythmias in subgroups stratified by the incidence of AMI during the index admission confirmed these results. However, after multivariable adjustment, sVT/VF was associated with a higher risk of long-term HF-related rehospitalization in HFmrEF patients experiencing AMI during the index admission.

Following the introduction of HFmrEF as a third and independent category of HF in 2016 and its update in 2021, clinical data regarding this cohort has slowly expanded, facilitating its progressive characterization. HFmrEF is currently thought to represent an “intermediate” between HFrEF and HFpEF [38]. Despite certain variations between studies, baseline characteristics typical for HFrEF but also for HFpEF have been observed, while the most consistent finding was the similarly high prevalence of IHD in HFmrEF and HFrEF (~60%) compared to HFpEF (~50%) [39,40,41]. This finding could have relevant implications for the incidence of ventricular arrhythmias, since IHD results in the formation of scar tissue, which can be a substrate for impaired electrical conduction and therefore ventricular arrhythmias [42,43]. Accordingly, IHD was the leading HF etiology in the present HFmrEF cohort in 58% of patients hospitalized with HFmrEF, which was predominantly observed in patients with nsVT and sVT/VF. Furthermore, logistic regression analysis confirmed that IHD was associated with the incidence of sustained ventricular tachyarrhythmias, corroborating the higher rates of sVT and VF in patients with IHD and AMI observed in previous research [6]. In addition, recent observational data suggest that patients suffering from HFmrEF might also benefit from therapies recommended for HFrEF, which led to the implementation of new recommendations for pharmacological treatments in recent international HF-guidelines [14,15,44,45,46,47,48]. However, there is still no data supporting the prognostic value of device-based therapies in patients with HFmrEF. 

Even though IHD and impaired systolic function are associated with the occurrence of ventricular tachyarrhythmias, prior RCT investigating the prognostic impact of ICD have typically focused on patients with LVEF <40% as their inclusion criteria [21,22,23,24,25]. Although studies have demonstrated that a large proportion of HFrEF patients transition to HFmrEF or even HFpEF as their LVEF improves over time [49,50,51]. This results in a discrepancy between patients’ LVEF (>35%) and the LVEF threshold suggested in the guideline recommendations (≤35%), which poses a challenge especially when considering primary prevention ICD implantation after an ischemic event or when generator replacement becomes necessary. Furthermore, Chatterjee et al. observed that the relative risk of SAD compared to non-SAD could already be elevated in patients with IHD and an LVEF of 40–49% [52]. Since there is data suggesting that a relevant proportion of patients with an LVEF >35% might suffer from ventricular tachyarrhythmias [6] and SCD [53], it is essential to establish a more personalized approach for individual risk assessment to properly guide decision-making regarding preventative measures such as the implantation of an ICD. This approach must consider additional relevant factors beyond LVEF such as HF etiology. In addition, parameters representative of cardiac filling pressure and especially ventricular wall stress could also aid in guiding the risk assessment of patients as these have been shown to be particularly related to the incidence of arrhythmias and are not adequately reflected by LVEF [54,55,56,57]. Ultimately, further research is warranted to identify predictors of ventricular tachyarrhythmias in specific patient cohorts to expand guideline recommendations. As there are currently no guideline recommendations for the use of ICD in patients with HFmrEF, the prevalence of ICD carriers in this cohort is low. Therefore, investigating ventricular tachyarrhythmias in this cohort is mainly possible through the documentation of clinically relevant or investigator-reported events, but not through large-scale registries selectively including patients with long-term continuous rhythm monitoring such as those with an ICD.

Just recently, Curtain et al. reported data from a post hoc analysis of the PARAGON-HF, TOPCAT, I-Preserve, and CHARM-Preserved trials. The authors investigated the association of VT and VF with mortality as well as different variables connected to the incidence of VT/VF in a pooled cohort of 2467 HFmrEF and 11142 HFpEF patients with a median LVEF of ~57% over a median follow-up of 1170 days. VT/VF was associated with all-cause and cardiovascular mortality. However, mortality was mainly driven by death from HF instead of SCD. Furthermore, a subgroup analysis of the HFmrEF cohort demonstrated higher rates of VT/VF in HFmrEF (2.0%) compared to HFpEF (0.9%), supporting prior study findings that reduced LVEF predicts ventricular arrhythmias in patients with HF [24,58]. Finally, Curtain et al. suggest that VT/VF is predominantly an indicator of disease severity rather than the risk of SCD [27]. The observation of higher mortality in patients with sVT/VF was not confirmed in our study. This discrepancy can likely be attributed to differences in the characteristics of the investigated cohorts. Especially pharmacologic treatment with beta-blockers, ACE-inhibitors/angiotensin receptor blockers (ARB), and statins was very common in the present study and has been shown to improve prognosis in patients with ventricular tachyarrhythmias [59,60]. Furthermore, Curtain et al. only performed an analysis of mortality for the mixed HFmrEF/HFpEF cohort and not selectively for HFmrEF patients, most likely due to the low incidence of ventricular tachyarrhythmias in the study cohort. However, it should be mentioned that there was a trend toward the statistical significance of sVT/VF in predicting all-cause mortality (*p* = 0.104) within the multivariable regression analysis presented in this study. Therefore, the absence of higher mortality could also be attributed to the fact that the study cohort might not have been powerful enough to identify a potential effect on prognosis, although this is one of the largest contemporary cohorts including patients with HFmrEF. Notably, IHD was very common in the present study and was associated with a favorable prognosis, which could further explain why similar mortality was observed in the group with sVT/VF and would support the improvements in the management of IHD observed within recent decades [61,62]. Additionally, previous investigations observed higher mortality in patients with IHD and VF compared to IHD and sVT [6]. Therefore, separate analyses of these groups might have yielded different results, which was not feasible due to the relatively low rate of ventricular tachyarrhythmias in the present study. Since the occurrence and prognostic impact of ventricular tachyarrhythmias might differ in patients with or without acute myocardial ischemia, sub-analyses stratified by the incidence of AMI during the index admission were performed (i.e., subgroups of HFmrEF patients with or without AMI). The results of these subgroup analyses primarily confirmed the findings observed in the entire study cohort, showing no association of nsVT or sVT/VF with long-term prognostic outcomes. However, in the subgroup of HFmrEF patients with AMI, the incidence of sVT/VF was associated with a significantly higher risk of long-term HF-related rehospitalization. This observation could indicate that the occurrence of sustained ventricular tachyarrhythmias in the setting of AMI might be a marker of more pathologically extensive myocardial damage, which may then result in a higher risk of decompensated heart failure even in patients with only mildly reduced LVEF.

While sustained ventricular tachyarrhythmias are inherently associated with cardiac pathology, nsVT can occur in apparently healthy hearts, as well as in a multitude of conditions associated with cardiac remodeling such as IHD, valvular heart disease, or certain cardiomyopathies [63,64]. It was observed that the incidence of nsVT increases in patients with hypertension-mediated LV hypertrophy (~12–28%) compared to those with hypertension but no significant hypertrophy (~8%) and healthy controls (~2%) [65,66]. Even though arterial hypertension was most common in the nsVT group (80%), there were no significant differences regarding septal hypertrophy between groups, which could potentially explain the low incidence of nsVT observed in the present study. In the setting of IHD and AMI, nsVT is a common finding, especially directly following an ischemic event. The persistence of nsVT beyond the initial days following such an event was thought to be associated with an increased risk of mortality, especially through SCD [67,68]. However, more recent studies from the beta-blocker and reperfusion era question the prognostic significance of nsVT, especially after adjustment for LVEF [33,69,70]. Notably, the observations of Gutierrez et al. and Mäkikallio et al. propose that nsVT could remain a relevant predictor of SCD in the subpopulation of patients with mildly reduced or even preserved LVEF [17,69]. However, the results of the present study do not support a trend toward an adverse prognostic impact of nsVT. Nonetheless, since nsVT was associated with higher NYHA functional class, its occurrence in patients with HF could be an indicator of advancing cardiac pathology resulting in this rhythm abnormality. Ultimately, the current state of evidence on nsVT as a prognostic marker of mortality remains ambiguous, and further research, especially in cohorts with an LVEF >40%, is necessary to identify when nsVT should be considered a relevant risk factor for sustained ventricular tachyarrhythmias and/or mortality.

### 4.1. Study Limitations

Due to the retrospective and single-center study design, results may be influenced by measured and unmeasured confounding and are not directly generalizable to other patient populations. Ventricular tachyarrhythmias were not exclusively detected by standardized ECG but by different diagnostic tools such as 12-lead ECG, ECG telemetry, ICD, or, in case of an unstable course or during resuscitation, by external defibrillator monitoring. Therefore, the true prevalence of ventricular tachyarrhythmias may be underestimated due to the lack of systematic rhythm monitoring in the index hospitalization as well as long-term rhythm monitoring in the follow-up period. Given that the incidence of ventricular tachyarrhythmias during the index hospitalization served as the primary parameter to define the groups for statistical analyses, this must be acknowledged as a significant confounding factor in the present study. Furthermore, inclusion was based on echocardiographically confirmed HFmrEF, which was typically performed during the index hospitalization following hemodynamic stabilization. Consequently, patients with ventricular tachyarrhythmias and very early death were not included in the present study, which may further contribute to a rather low rate of ventricular tachyarrhythmias within the present registry. In addition, causes of death beyond those occurring within the index hospitalization were not available for the present study. In general, the sample size of patients experiencing ventricular tachyarrhythmias was relatively limited and might therefore have been underpowered to detect potential adverse implications on long-term prognosis. Finally, patients with mildly reduced ejection fraction in the context of acute cardiac events (e.g., ischemia or rhythm disorders) might experience recovery of LVEF after their index hospitalization, leading to their transition out of the HFmrEF category during the follow-up period.

### 4.2. Conclusions

Considering the results of the present study, the occurrence of ventricular tachyarrhythmias remains most common in the clinical setting of myocardial ischemia (i.e., IHD and AMI). Ventricular tachyarrhythmias were not associated with long-term prognostic endpoints. The absence of an adverse prognostic impact might be explained by the more extensive pharmacological treatment in the nsVT and sVT/VF cohorts as well as the general improvements regarding the medical management of IHD observed in the last decades. Sustained and non-sustained ventricular tachyarrhythmias in patients with HFmrEF might be considered as a marker of disease severity that could indicate the need for closer patient monitoring and more comprehensive medical management to avoid adverse prognostic implications. 

## Figures and Tables

**Table 1:** Baseline characteristics.

	No Ventricular Arrhythmia (*n* = 2088)		nsVT (*n* = 44)		sVT/VF (*n* = 52)		*p*-Value across Groups	*p*-Value None vs. nsVT	*p*-Value None vs. sVT/VF	*p*-Value nsVT vs. sVT/VF
**Age**, median (IQR)	76 (64–83)		74 (68–82)		68 (56–77)		**0.003**	0.610	**0.001**	0.034 *
**Male sex**, n (%)	1339	(64.1)	31	(70.5)	40	(76.9)	0.116	-	-	-
**Body mass index**, kg/m^2^, median (IQR)	26.6 (23.8–30.7)		26.5 (24.6–30.8)		26.7 (24.0–32.2)		0.771	-	-	-
**SBP**, mmHg, median (IQR)	143 (125–163)		141 (115–150)		133 (119–152)		**0.050**	0.187	0.037 *	0.581
**DBP**, mmHg, median (IQR)	80 (69–90)		75 (67–89)		75 (64–87)		0.368	-	-	-
**Heart rate**, bpm, median (IQR)	80 (68–94)		84 (70–100)		82 (71–99)		0.370	-	-	-
**Medical history**, n (%)										
Coronary artery disease	856	(41.0)	20	(45.5)	20	(38.5)	0.779	-	-	-
Prior myocardial infarction	495	(23.7)	9	(20.5)	17	(32.7)	0.281	-	-	-
Prior PCI	583	(27.9)	13	(29.5)	16	(30.8)	0.880	-	-	-
Prior CABG	202	(9.7)	7	(15.9)	5	(9.6)	0.387	-	-	-
Prior valvular surgery	91	(4.4)	3	(6.8)	2	(3.8)	0.719	-	-	-
Congestive heart failure	705	(33.8)	17	(38.6)	19	(36.5)	0.734	-	-	-
Decompensated heart failure < 12 months	228	(10.9)	5	(11.4)	5	(9.6)	0.952	-	-	-
Prior sVT/VF	53	(2.5)	5	(11.4)	8	(15.4)	**0.001**	**0.001**	**0.001**	0.566
Prior ICD	33	(1.6)	2	(4.5)	7	(13.5)	**0.001**	0.126	**0.001**	0.135
Prior sICD	9	(0.4)	0	(0.0)	0	(0.0)	0.812	-	-	-
Prior CRT-D	29	(1.4)	1	(2.3)	2	(3.8)	0.313	-	-	-
Indication for (s)ICD/CRT-D										
Primary prevention	54	(2.6)	1	(2.3)	4	(7.7)	**0.001**	0.033 *	**0.001**	0.289
Secondary prevention	17	(0.8)	2	(4.5)	5	(9.6)				
Prior pacemaker	193	(9.2)	4	(9.1)	3	(5.8)	0.692	-	-	-
Chronic kidney disease	658	(31.5)	11	(25.0)	10	(19.2)	0.114	-	-	-
Peripheral artery disease	239	(11.4)	6	(13.6)	8	(15.4)	0.621	-	-	-
Stroke	320	(15.3)	5	(11.4)	6	(11.5)	0.586	-	-	-
Liver cirrhosis	45	(2.2)	1	(2.3)	1	(1.9)	0.992	-	-	-
Malignancy	329	(15.8)	3	(6.8)	3	(5.8)	0.041	-	0.049 *	-
COPD	253	(12.1)	7	(15.9)	3	(5.8)	0.227	-	-	-
**Cardiovascular risk factors**, n (%)										
Arterial hypertension	1630	(78.1)	35	(79.5)	37	(71.2)	0.478	-	-	-
Diabetes mellitus	773	(37.0)	10	(22.7)	16	(30.8)	0.102	-	-	-
Hyperlipidemia	621	(29.7)	20	(45.5)	21	(40.4)	**0.022**	0.024 *	0.098	0.617
Smoking										
Current	384	(18.4)	11	(25.0)	11	(21.2)	0.426	-	-	-
Former	371	(17.8)	8	(18.2)	11	(21.2)		-	-	-
Family history	186	(8.9)	7	(15.9)	8	(15.4)	0.084	-	-	-
**Entry criteria**, n (%)										
Acute coronary syndrome	525	(25.1)	16	(36.4)	28	(53.8)	**0.001**	0.091	**0.001**	0.087
Chronic coronary syndrome	274	(13.1)	0	(0.0)	5	(9.6)	**0.028**	**0.010**	0.458	0.035 *
Rhythm disorders	469	(22.5)	12	(27.3)	15	(28.9)	0.425	-	-	-
Ventricular tachyarrhythmia	0	(0.0)	4	(9.1)	11	(21.2)	**0.001**	**0.001**	**0.001**	0.105
Acute decompensated heart failure	330	(15.8)	8	(18.2)	2	(3.8)	0.056	-	0.019 *	0.022 *
Valvular disease	170	(8.1)	4	(9.1)	0	(0.0)	0.097	-	0.032 *	0.026 *
Pulmonary embolism	28	(1.3)	1	(2.3)	0	(0.0)	0.606	-	-	-
Cardiomyopathy	96	(4.6)	2	(4.5)	0	(0.0)	0.286	-	-	-
Elective cardiac procedure	31	(1.5)	0	(0.0)	0	(0.0)	0.485	-	-	-
Others	165	(7.9)	1	(2.3)	1	(3.8)	0.219	-	-	-
**Comorbidities at index hospitalization**, n (%)										
Acute coronary syndrome										
Unstable angina	97	(4.6)	1	(2.3)	1	(1.9)	0.497	-	-	-
STEMI	150	(7.2)	11	(25.0)	15	(28.8)	**0.001**	**0.001**	**0.001**	0.673
NSTEMI	257	(12.3)	5	(11.4)	12	(23.1)	0.067	-	-	-
Acute decompensated heart failure	464	(22.2)	11	(25.0)	9	(17.3)	0.631	-	-	-
Atrial fibrillation	882	(42.2)	21	(47.7)	13	(25.0)	**0.033**	0.466	**0.013**	0.020 *
Cardiopulmonary resuscitation	25	(1.2)	1	(2.3)	27	(51.9)	**0.001**	0.520	**0.001**	**0.001**
Out-of-hospital	7	(0.3)	0	(0.0)	15	(28.8)	**0.001**	0.700	**0.001**	**0.001**
In-hospital	18	(0.9)	1	(2.3)	12	(23.1)	**0.001**	0.324	**0.001**	**0.003**
Stroke	295	(14.1)	2	(4.5)	1	(1.9)	**0.008**	0.069	**0.012**	0.462
**Implantation of cardiac devices during index hospitalization**, n (%)										
ICD	3	(0.014)	1	(2.3)	4	(7.7)	**0.001**	**0.001**	**0.001**	0.234
sICD	2	(0.096)	2	(4.5)	5	(9.6)	**0.001**	**0.001**	**0.001**	0.341
CRT-D	1	(0.005)	0	(0.0)	0	(0.0)	0.977	-	-	-
**Medication on admission**, n (%)										
ACE-inhibitor	748	(35.8)	9	(20.5)	18	(34.6)	0.107	-	-	-
ARB	468	(22.4)	10	(22.7)	11	(21.2)	0.976	-	-	-
Beta-blocker	1183	(56.7)	23	(52.3)	28	(53.8)	0.783	-	-	-
Aldosterone antagonist	197	(9.4)	3	(6.8)	6	(11.5)	0.733	-	-	-
ARNI	19	(0.9)	0	(0.0)	0	(0.0)	0.644	-	-	-
SGLT2-inhibitor	41	(2.0)	2	(4.5)	2	(3.8)	0.322	-	-	-
Loop diuretics	794	(38.0)	12	(27.3)	15	(28.8)	0.145	-	-	-
Statin	939	(45.0)	23	(52.3)	23	(44.2)	0.624	-	-	-
ASA	704	(33.7)	14	(31.8)	17	(32.7)	0.955	-	-	-
P2Y12-inhibitor	202	(9.7)	4	(9.1)	5	(9.6)	0.992	-	-	-
DOAC	495	(23.7)	15	(34.1)	10	(19.2)	0.204	-	-	-
Vitamin K antagonist	180	(8.6)	2	(4.5)	3	(5.8)	0.491	-	-	-

ACE, angiotensin-converting enzyme; ARB, angiotensin receptor blocker; ARNI, angiotensin receptor neprilysin inhibitor; ASA, acetylsalicylic acid; CABG, coronary artery bypass grafting; CKD, chronic kidney disease; COPD, chronic obstructive pulmonary disease; CRT-D, cardiac resynchronization therapy with defibrillator; DBP, diastolic blood pressure; DOAC, directly acting oral anticoagulant; IQR, interquartile range; (N)STEMI, non-ST-segment elevation myocardial infarction; nsVT, non-sustained ventricular tachycardia; SBP, systolic blood pressure; SGLT2, sodium-glucose linked transporter 2; (s) ICD, (subcutaneous) implantable cardioverter defibrillator; sVT, sustained ventricular tachycardia; VF, ventricular fibrillation. Level of significance *p* ≤ 0.05. Bold type indicates statistical significance. * According to the Bonferroni method, the α-significance level for the pairwise comparisons was adjusted to *p* ≤ 0.05/3 = 0.017.

**Table 2:** Heart failure-related and procedural data.

	No Ventricular Arrhythmia (*n* = 2088)		nsVT (*n* = 44)		sVT/VF (*n* = 52)		*p*-Value across Groups	*p*-Value None vs. nsVT	*p*-Value None vs. sVT/VF	*p*-Value nsVT vs. sVT/VF
**Heart failure etiology**, n (%)							**0.006**	0.221	**0.003**	0.199
Ischemic heart disease	1181	(56.6)	31	(70.5)	46	(88.5)				
Non-ischemic cardiomyopathy	140	(6.7)	6	(13.6)	3	(5.8)				
Hypertensive cardiomyopathy	177	(8.5)	1	(2.3)	0	(0.0)				
Congenital heart disease	4	(0.2)	0	(0.0)	0	(0.0)				
Valvular heart disease	94	(4.5)	2	(4.5)	0	(0.0)				
Tachycardia associated	127	(6.1)	1	(2.3)	0	(0.0)				
Tachymyopathy	38	(1.8)	0	(0.0)	0	(0.0)				
Pacemaker-induced cardiomyopathy	19	(0.9)	0	(0.0)	0	(0.0)				
Unknown	346	(16.6)	3	(6.8)	3	(5.8)				
**NYHA functional class**, n (%)										
I/II	1515	(72.6)	30	(68.2)	40	(76.9)	0.073	-	-	-
III	397	(19.0)	9	(20.5)	4	(7.7)				
IV	176	(8.4)	5	(11.4)	8	(15.4)				
**Echocardiographic data**										
LVEF, %, median (IQR)	45 (45–47)		45 (44–47)		45 (43–46)		0.362	-	-	-
IVSd, median (IQR)	12 (11–13)		11 (10–13)		12 (11–13)		0.306	-	-	-
LVEDD, mm, median (IQR)	49 (44–54)		50 (47–56)		50 (47–54)		0.073	-	-	-
TAPSE, mm, median (IQR)	20 (17–23)		21 (18–23)		20 (18–23)		0.480	-	-	-
LA diameter, mm, median (IQR)	42 (37–47)		40 (36–52)		41 (35–46)		0.725	-	-	-
LA surface, cm^2^, median (IQR)	22 (17–26)		20 (17–24)		20 (17–26)		0.577	-	-	-
E/A, median (IQR)	0.8 (0.6–1.2)		0.8 (0.7–1.2)		0.8 (0.7–1.1)		0.800	-	-	-
E/E‘, median (IQR)	9.3 (6.5–14.0)		8.8 (5.3–11.0)		10.5 (7.5–12.4)		0.602	-	-	-
Diastolic dysfunction, n (%)	1515	(72.6)	27	(61.4)	32	(61.5)	0.060	-	-	-
Moderate–severe aortic stenosis, n (%)	207	(9.9)	4	(9.1)	3	(5.8)	0.603	-	-	-
Moderate–severe aortic regurgitation, n (%)	79	(3.8)	2	(4.5)	3	(5.8)	0.741	-	-	-
Moderate–severe mitral regurgitation, n (%)	252	(12.1)	3	(6.8)	7	(13.5)	0.540	-	-	-
Moderate–severe tricuspid regurgitation, n (%)	336	(16.1)	4	(9.1)	4	(7.7)	0.123	-	-	-
VCI, mm, median (IQR)	20 (15–25)		17 (13–31)		18 (14–22)		0.459	-	-	-
Aortic root, mm, median (IQR)	33 (30–36)		32 (30–38)		34 (30–35)		0.927	-	-	-
**Coronary angiography**, n (%)	826	(39.6)	28	(63.6)	46	(88.5)	**0.001**	**0.001**	**0.001**	**0.004**
No evidence of coronary artery disease	166	(20.1)	3	(10.7)	6	(13.0)	**0.009**	**0.002**	0.594	0.033 *
1-vessel disease	146	(17.1)	13	(46.4)	7	(15.2)				
2-vessel disease	177	(21.4)	4	(14.3)	11	(23.9)				
3-vessel disease	337	(40.8)	8	(28.6)	22	(47.8)				
CABG	63	(7.6)	5	(17.9)	5	(10.9)	0.117	-	-	-
Chronic total occlusion	95	(11.5)	2	(7.1)	16	(34.8)	**0.001**	0.475	**0.001**	**0.007**
PCI, n (%)	431	(52.2)	17	(60.7)	33	(71.7)	**0.026**	0.374	**0.010**	0.326
Sent to CABG, n (%)	50	(6.1)	0	(0.0)	1	(2.2)	0.227	-	-	-
**Baseline laboratory values**, median (IQR)										
Potassium, mmol/L	3.9 (3.6–4.2)		3.8 (3.6–4.1)		3.9 (3.7–4.2)		0.780	-	-	-
Sodium, mmol/L	139 (137–141)		140 (138–142)		140 (138–141)		0.227	-	-	-
Creatinine, mg/dL	1.07 (0.86–1.46)		0.99 (0.85–1.46)		0.97 (0.80–1.26)		0.054	-	-	-
eGFR, mL/min/1.73 m^2^	65 (45–85)		79 (47–92)		79 (56–95)		**0.004**	0.076	**0.004**	0.508
Hemoglobin, g/dL	12.4 (10.4–14.0)		12.7 (10.6–13.8)		12.3 (10.4–14.2)		0.895	-	-	-
WBC count, ×10^9^/L	8.16 (6.42–10.10)		8.97 (6.78–10.17)		8.89 (7.83–10.52)		**0.030**	0.429	**0.011**	0.291
Platelet count, ×10^9^/L	225 (178–284)		217 (153–285)		269 (204–349)		**0.003**	0.387	**0.001**	**0.007**
HbA1c, %	5.9 (5.5–6.8)		5.6 (5.5–6.3)		5.9 (5.4–6.5)		0.274	-	-	-
LDL-cholesterol, mg/dL	99 (74–127)		84 (73–128)		81 (59–119)		0.100	-	-	-
HDL-cholesterol, mg/dL	42 (34–52)		44 (34–50)		37 (29–43)		**0.032**	0.836	**0.009**	0.048 *
C-reactive protein, mg/L	13.1 (3.4–43.7)		12.7 (3.2–36.6)		17.4 (4.6–47.0)		0.652	-	-	-
NT-pro BNP, pg/mL	2682 (994–6811)		2773 (1026–8560)		1971 (1227–3500)		0.802	-	-	-
NT-pro BNP (eGFR corrected), pg/mL	1630 (652–3428)		2300 (674–4848)		1665 (1080–4267)		0.502	-	-	-
Cardiac troponin I, µg/L	0.03 (0.02–0.16)		0.03 (0.02–3.19)		0.03 (0.02–2.01)		**0.001**	0.146	**0.001**	0.325
**Medication at discharge**, n (%)										
ACE-inhibitor	1006	(49.9)	19	(43.2)	33	(68.8)	**0.023**	0.380	**0.010**	**0.013**
ARB	479	(23.7)	11	(25.0)	9	(18.8)	0.707	-	-	-
Beta-blocker	1548	(76.7)	39	(88.6)	48	(100.0)	**0.001**	0.064	**0.001**	**0.016**
Aldosterone antagonist	279	(13.8)	5	(11.4)	12	(25.0)	0.078	-	-	-
ARNI	24	(1.2)	1	(2.3)	0	(0.0)	0.600	0.516	0.447	0.294
SGLT2-inhibitor	80	(4.0)	1	(2.3)	3	(6.3)	0.612	0.567	0.426	0.350
Loop diuretics	981	(48.6)	17	(38.6)	20	(41.7)	0.275	0.189	0.340	0.767
Statin	1367	(67.8)	36	(81.8)	39	(81.3)	**0.021**	0.048 *	0.048 *	0.944
Digitalis	100	(5.0)	3	(6.8)	0	(0.0)	0.241	-	-	-
Amiodarone	50	(2.0)	2	(4.5)	6	(12.5)	**0.001**	0.387	**0.001**	0.176
ASA	1003	(49.7)	25	(56.8)	35	(72.9)	**0.004**	0.352	**0.001**	0.105
P2Y12-inhibitor	611	(30.3)	22	(50.0)	35	(72.9)	**0.001**	**0.005**	**0.001**	0.024 *
DOAC	660	(32.7)	17	(38.6)	13	(27.1)	0.498	-	-	-
Vitamin K antagonist	145	(7.2)	2	(4.5)	3	(6.3)	0.775	-	-	-

ACE, angiotensin-converting enzyme; ARB, angiotensin receptor blocker; ARNI, angiotensin receptor neprilysin inhibitor; ASA, acetylsalicylic acid; CABG, coronary artery bypass grafting; DOAC, directly acting oral anticoagulant; eGFR, estimated glomerular filtration rate; HbA1c, glycated hemoglobin; HDL, high-density lipoprotein; IQR, interquartile range; IVSd, interventricular septum in diastole; LA, left atrial; LDL, low-density lipoprotein; LVEDD, left ventricular end-diastolic diameter; LVEF, left ventricular ejection fraction; nsVT, non-sustained ventricular tachycardia; NT-pro BNP, N-terminal prohormone of brain natriuretic peptide; NYHA, New York Heart Association; PCI, percutaneous coronary intervention; sVT, sustained ventricular tachycardia; TAPSE, tricuspid annular plane systolic excursion; VCI, vena cava inferior; VF, ventricular fibrillation; WBC, white blood cells. Level of significance *p* ≤ 0.05. Bold type indicates statistical significance. * According to the Bonferroni method, the α-significance level for the pairwise comparisons was adjusted to *p* ≤ 0.05/3 = 0.017.

**Table 3:** Specific data regarding sustained ventricular arrhythmias (sVT and VF).

	sVT/VF (*n* = 52)	
Arrhythmia *, n (%)		
sVT	16	(30.8)
VF	27	(51.9)
Both	9	(17.3)
**Time of arrhythmia**, n (%)		
On admission	30	(57.7)
In-hospital	22	(42.3)
**Symptoms**, n (%)		
None	12	(23.1)
Dyspnea	3	(5.8)
Angina pectoris	13	(25.0)
Syncope	11	(21.2)
CPR	26	(50.0)
**Arrhythmia termination**, n (%)		
Spontaneous	2	(3.8)
Amiodarone	16	(30.8)
Cardioversion	5	(9.6)
Defibrillation	40	(76.9)
Termination refused (DNR)	1	(1.9)

CPR, cardiopulmonary resuscitation; DNR, do-not-resuscitate order; sVT, sustained ventricular tachycardia; VF, ventricular fibrillation. * Two patients suffered from Torsades de pointes.

**Table 4:** Follow-up data, primary and secondary endpoints.

	No Ventricular Arrhythmia (n = 2088)		nsVT (n = 44)		sVT/VF (n = 52)		HR None vs. nsVT	95% CI None vs. nsVT	*p* Value None vs. nsVT	HR None vs. sVT/VF	95% CI None vs. sVT/VF	*p* Value None vs. sVT/VF	*p* Value nsVT vs. sVT/VF
**Primary endpoint**, n (%)													
All-cause mortality, at 30 months	657	(31.5)	11	(25.0)	15	(28.8)	0.760	0.419–1.380	0.367	0.928	0.556–1.549	0.776	-
**Secondary endpoints**, n (%)													-
All-cause mortality, in-hospital	71	(3.4)	0	(0.0)	4	(7.7)	-	-	-	2.092	0.763–5.737	0.151	-
Cardiovascular mortality, in-hospital	31	(1.5)	-	(-)	4	(7.7)	-	-	-	4.587	1.618–13.003	**0.004**	-
Non-cardiovascular mortality, in-hospital	40	(1.9)	-	(-)	-	(-)	-	-	-	-	-	-	-
Malignancy-associated mortality, in-hospital	14	(0.7)	-	(-)	-	(-)	-	-	-	-	-	-	-
All-cause mortality, at 12 months	448	(21.5)	8	(18.2)	10	(19.2)	0.811	0.403–1.631	0.557	0.914	0.488–1.711	0.779	-
Heart failure-related rehospitalization, at 30 months	266	(13.2)	6	(13.6)	7	(14.6)	1.046	0.466–2.349	0.914	1.112	0.525–2.354	0.782	-
Cardiac rehospitalization, at 30 months	435	(21.6)	12	(27.3)	15	(31.3)	1.338	0.754–2.375	0.319	1.542	0.921–2.580	0.099	-
Revascularization, at 30 months	133	(6.6)	4	(9.1)	5	(10.4)	1.407		0.501	1.637	0.670–3.997	0.279	-
Acute myocardial infarction, at 30 months	60	(3.0)	1	(2.3)	3	(6.3)	0.770	0.107–5.553	0.795	2.145	0.673–6.840	0.197	-
Stroke, at 30 months	56	(2.8)	0	(0.0)	1	(2.1)	-	-	-	0.758	0.105–5.472	0.783	-
MACCE, at 30 months	805	(38.6)	15	(34.1)	21	(40.4)	0.857	0.514–1.427	0.552	1.112	0.721–1.715	0.631	-
**Follow-up data**, median (IQR)													
Hospitalization time, days	9 (5–15)		9 (6–17)		11 (7–16)		-	-	0.585	-	-	0.053	0.282
ICU time, days	0 (0–1)		1 (0–2)		2 (1–5)		-	-	**0.001**	-	-	**0.001**	**0.001**
Follow-up time, days	900 (365–1650)		1103 (358–1504)		846 (339–1748)		-	-	0.649	-	-	0.713	0.944

CI, confidence interval; COPD, chronic obstructive pulmonary disease; HR, hazard ratio; ICU, intensive care unit; IQR, interquartile range; MACCE, major adverse cardiac and cerebrovascular events; nsVT, non-sustained ventricular tachycardia; sVT, sustained ventricular tachycardia; VF, ventricular fibrillation. Level of significance *p* ≤ 0.05. Bold type indicates statistical significance.

## Data Availability

The datasets used and/or analyzed during the current study are available from the corresponding author upon reasonable request.

## Supplementary Materials

The following supporting information can be downloaded at https://www.mdpi.com/article/10.3390/jcm13092665/s1, Supplementary Table S1: Correlation of all parameters used in multivariable regression analyses within the entire study cohort; Supplementary Table S2: Collinearity diagnostics of parameters used in multivariable regression analyses; Supplementary Table S3: Univariable Cox regression analyses of all parameters used in multivariable regression analyses.
