# Supplementary material for: Characteristics Associated with Ventricular Tachyarrhythmias and Their Prognostic Impact in Heart Failure with Mildly Reduced Ejection Fraction
Source: J Clin Med. 2024 May 1;13(9):2665. doi: 10.3390/jcm13092665 (PMC11084292; doi:10.3390/jcm13092665)
Supplement: Supplementary file 1 [file jcm-13-02665-s001.zip › jcm-2949634-supplementary.pdf]

Supplemental Table S1. Correlation of all parameters used in multivariable regression analyses within the entire study cohort.

**Spearman's ρ**	**Age** (years)	**BMI** (kg/m^2)	**Prior congestive heart failure**	**Chronic kidney disease**	**Diabetes**	**Acute myocardial infarction**	**Atrial fibrillation**	**NYHA functional class**	**Ischemic heart disease**	**nsVT**	**sVT/VF**
**Age** (years)	-	-0.198	0.122	0.301	0.086	-0.108	0.305	0.180	0.033	-0.013	-0.073
**BMI** (kg/m^2)	-0.167	-	0.009	-0.092	0.052	0.097	-0.080	-0.077	0.164	0.017	0.040
**Prior congestive heart failure**	0.122	0.015	-	0.260	0.090	-0.161	0.187	0.276	0.124	0.007	0.009
**Chronic kidney disease**	0.301	-0.021	0.260	-	0.153	-0.139	0.151	0.241	0.032	-0.023	-0.040
**Diabetes**	0.086	0.236	0.090	0.153	-	0.009	0.019	0.114	0.109	-0.046	-0.019
**Acute myocardial infarction**	-0.108	0.023	-0.161	-0.139	0.009	-	-0.210	-0.008	0.436	0.058	0.121
**Atrial fibrillation**	0.305	-0.030	0.187	0.151	0.019	-0.210	-	0.170	-0.125	0.013	-0.054
**NYHA functional class**	0.180	-0.001	0.276	0.241	0.114	-0.008	0.170	-	0.101	0.041	0.009
**Ischemic heart disease**	0.033	0.079	0.124	0.032	0.109	**0.436**	-0.125	0.101	-	0.051	0.098
**nsVT**	-0.013	0.012	0.007	-0.023	-0.046	0.058	0.013	0.041	0.051	-	0.097
**sVT/VF**	-0.073	0.015	0.009	-0.040	-0.019	0.121	-0.054	0.009	0.098	0.097	-

BMI, body mass index; nsVT, non-sustained ventricular tachycardia; NYHA, New York Heart Association; sVT, sustained ventricular tachycardia; VF, ventricular fibrillation.

Supplemental Table S2. Collinearity diagnostics of parameters used in multivariable regression analyses (dependent variable: 30-month all-cause mortality).

	VIF	Tolerance
**Age** (years)	1.272	0.786
**BMI** (kg/m^2)	1.111	0.900
**Prior congestive heart failure**	1.196	0.836
**Chronic kidney disease**	1.195	0.837
**Diabetes**	1.108	0.903
**Acute myocardial infarction**	1.367	0.732
**Atrial fibrillation**	1.185	0.844
**NYHA functional class**	1.150	0.870
**Ischemic heart disease**	1.333	0.750
**nsVT**	1.019	0.981
**sVT/VF**	1.034	0.967

BMI, body mass index; nsVT, non-sustained ventricular fibrillation; NYHA, New York Heart Association; sVT, sustained ventricular fibrillation; VF, ventricular fibrillation; VIF, variance-inflation-factor.

Supplemental Table S3. Univariable Cox regression analyses of all parameters used in multivariable regression analyses.

	30-month all-cause mortality		
**Variables**	**HR**	**95% CI**	**p-value**
**Age** (years)	1.052	1.045-1.060	**0.001**
**BMI** (kg/m^2)	0.939	0.923-0.956	**0.001**
**Prior congestive heart failure**	1.546	1.328-1.799	**0.001**
**Chronic kidney disease**	2.518	2.167-2.926	**0.001**
**Diabetes**	1.277	1.097-1.487	**0.002**
**Acute myocardial infarction**	0.615	0.498-0.761	**0.001**
**Atrial fibrillation**	1.856	1.597-2.158	**0.001**
**NYHA functional class** (I-IV)	1.347	1.255-1.447	**0.001**
**Ischemic heart disease**	0.786	0.676-0.914	**0.002**
**nsVT**	0.816	0.471-1.413	0.468
**sVT/VF**	0.933	0.559-1.557	0.791

BMI, body mass index; CI, confidence interval; HR, hazard ratio; nsVT, non-sustained ventricular fibrillation; NYHA, New York Heart Association; sVT, sustained ventricular fibrillation; VF, ventricular fibrillation. Level of significance p≤0.05. Bold type indicates statistical significance.
